# Correction to “Ephedrine for Reducing Onset Time of Rocuronium in Elderly Patients: A Randomised Study”

**DOI:** 10.1111/aas.70392

**Published:** 2026-11-11

M. Vested , M. F. Hovind , T. Nielsen , et al., “Ephedrine for Reducing Onset Time of Rocuronium in Elderly Patients: A Randomised Study,” Acta Anaesthesiologica Scandinavica 70, no. 10 (2026): e70344, 10.1111/aas.70344.PMC1361516242791593

In the published version of the article, the calculation of the difference in occurrence of all types of cardiac arrhythmias in the Abstract, Results section, and Table 2 were incorrect. The correct information is provided below.

In the Abstract, under Results section, the text:

Cardiac arrythmias occurred more often in the ephedrine group compared to the placebo group (*p* < 0.001).

This should have read as:

No difference was found in occurrence of all types of cardiac arrhythmias in the ephedrine group compared to the placebo group (*p* = 0.12); however, sinus tachycardia occurred more often in the ephedrine group (*p* < 0.001).

In the Results section, fifth paragraph:

Cardiac arrythmias within 15 minutes after administration of the study drug occurred more often in the ephedrine group compared to the placebo group (*p* < 0.001) (Table 2).

This should have read as:

No difference was found in occurrence of all types of cardiac arrhythmias within 15 minutes after administration of the study drug comparing patients administered ephedrine with placebo (*p* = 0.012) (Table 2). However, sinus tachycardia occurred more often among patients administered ephedrine (*p* < 0.001), and sinus bradycardia occurred more often among patients administered placebo (*p* = 0.004) (Table 2).

The correct Table 2 is:

**TABLE 2:** Outcomes in patients 80 years or above randomised to either ephedrine 0.15 mg kg^−1^ or placebo (saline) before administration of rocuronium.

	Ephedrine	Placebo	*p*	Difference with 95% CI
*n*	41	39		
Time to TOF 0 (s)	134 (IQR, 107 to 180)	139 (IQR, 121 to 185)	0.60***	9 (−14 to 31)
Time to TOF 90 (min)	87 (35) *n* = 6	83 (32) *n* = 8	0.8**	4 (−34 to 44)
kMAP before induction (mmHg)	99 (12)	98 (12)		
MAP after intubation (mmHg)	103 (25)	92 (20)		
Change in MAP (mmHg)	4 (21)	−6 (22)	0.03**	10 (1 to 20)
Cardiac arrythmia				
Sinus bradycardia (SB)	0	7 (18%)	0.004	−18 (−31 to −6)
Sinus tachycardia (ST)	16 (39%)	1 (3%)	< 0.001	36 (20 to 52)
Sinus tachycardia and bradycardia	0	1 (2%)		
Occurrence of hoarseness 24 h postoperatively				
	9 (22%)	11 (28%)	0.73*	
72 h postoperatively				
	3 (33%)	4 (40%)	0.55*	
Occurrence of sore throat 24 h postoperatively				
	7 (17%)	5 (13%)	0.73*	
Missing	*n* = 2 (5%)	*n* = 1 (2%)		
72 h postoperatively				
	4 (57%)	0	0.06*	
Intubation difficulty scale				
Not excellent	16 (39%)	16 (42%)	0.78*	
Excellent	25 (61%)	22 (58%)		
Fuchs‐Buder				
Not excellent	12 (29%)	18 (47%)	0.10*	
Excellent	29 (71%)	20 (53%)		

We apologize for these errors.

